# Genome sequence of a multidrug-resistant Corynebacterium striatum isolated from bloodstream infection from a nosocomial outbreak in Rio de Janeiro, Brazil

**DOI:** 10.1590/0074-02760180051

**Published:** 2018-07-10

**Authors:** Juliana Nunes Ramos, Izabel dos Santos Rodrigues, Paulo Victor Pereira Baio, João Flávio Carneiro Veras, Rommel Thiago Jucá Ramos, Luis GC Pacheco, Vasco Ariston Azevedo, Raphael Hirata, Michel Abanto Marín, Ana Luiza de Mattos-Guaraldi, Verônica Viana Vieira

**Affiliations:** 1 Fundação Oswaldo Cruz-Fiocruz, Instituto Nacional de Controle de Qualidade em Saúde, Rio de Janeiro, RJ, Brasil; 2 Universidade do Estado do Rio de Janeiro, Faculdade de Ciências Médicas, Laboratório de Difteria e Corinebactérias de Importância Clínica, Rio de Janeiro, RJ, Brasil; 3 Fundação Oswaldo Cruz-Fiocruz, Instituto Oswaldo Cruz, Laboratório Interdisciplinar de Pesquisas Médicas, Rio de Janeiro, RJ, Brasil; 4 Universidade Federal do Pará, Centro de Genômica e Biologia de Sistemas, Belém, PA, Brasil; 5 Universidade Federal da Bahia, Instituto de Ciências da Saúde, Salvador, BA, Brasil; 6 Universidade Federal de Minas Gerais, Instituto de Ciências Biológicas, Belo Horizonte, MG, Brasil; 7 University of La Frontera, Scientific and Technological Bioresource Nucleus, Temuco, Chile

**Keywords:** multidrug-resistant, Corynebacterium striatum, bloodstream infection, CRISPR-Cas, spaDEF cluster

## Abstract

Multidrug-resistant (MDR) Corynebacterium striatum has been cited with increased frequency as pathogen of nosocomial infections. In this study, we report the draft genome of a C. striatum isolated from a patient with bloodstream infection in a hospital of Rio de Janeiro, Brazil. The isolate presented susceptibility only to tetracycline, vancomycin and linezolid. The detection of various antibiotic resistance genes is fully consistent with previously observed multidrug-resistant pattern in Corynebacterium spp. A large part of the pTP10 plasmid of MDR C. striatum M82B is present in the genome of our isolate. A SpaDEF cluster and seven arrays of CRISPR-Cas were found.

Corynebacterium striatum is a Gram-positive rod, constituent of the normal microbiota of the skin and mucous membranes, however, potentially pathogenic under specific circumstances, including infections of patients with chronic diseases and the use of invasive procedures^.(^ ^1^ ^,^ ^2^ This microorganism has been responsible for a variety of invasive infections, such as bacteremia,^3^ endocarditis,^4^ osteomyelitis,^5^ and others. C. striatum isolates also emerged as pathogens related to nosocomial outbreaks in several countries, such as Spain,^6^ Brazil,^7^ Belgium,^8^ Japan^9^ and Tunisia.^10^

Here, we present the draft genome of C. striatum 2308 isolated from blood in pure culture, of a male patient in August, 2011 attended at University Hospital Pedro Ernesto, Rio de Janeiro, Brazil. This isolate was deposited at Coleção de Bactérias do Ambiente e Saúde (CBAS/FIOCRUZ) under deposit number CBAS 614. The consent to participate was not required because the investigated isolate was taken as a part of standard care (diagnostic purposes). This study was developed in compliance with the Brazilian Government’s Ethical Guidelines for research involving human beings (resolution of the National Health Council/Ministry of Health) and approved by the ethical research committee of HUPE/UERJ (CAAE: 01247512.3.0000.5259.

Genotyping studies by pulsed-field gel electrophoresis (PFGE) classified the isolate as PFGE I profile, revealing the permanence of this clone^7^ in the nosocomial environment as invasive clone (data not shown). This isolate was submitted for an antimicrobial susceptibility test by minimum inhibitory concentration (MIC) using E-test strips (AB Biodisk, Sweden) on standard Mueller Hinton agar containing 5% sheep blood. Nine antimicrobial compounds were tested: penicillin, ciprofloxacin, levofloxacin, gentamicin, vancomycin, clindamycin, erythromycin, tetracycline and linezolid.^11^

Whole genome sequencing of C. striatum 2308 isolate was performed using Illumina HiSeq 2500 sequencer (Illumina Inc, USA). A library was constructed with the Nextera XT DNA Library Preparation Kit (Illumina). The sequencing process rendered 8108300 reads of 100 bp, which represents a coverage of 254X. The reads were assembled de novo using the CLC Genomics Workbench 6.5 (Available from: http://www.clcbio.com/products/clc-main-workbench/) and MIRA 3.9.18 (Available from: http://sourceforge.net/projects/mira-assembler/). The curation to reduce The assembly produced the gaps was done with the Lasergene v.11 Suite (DNASTAR). 73 contigs with total de 3.003,571 pb, N50 of 142 kb; the longest contig is 551 kb. The contigs were annotaded using NCBI Prokaryotic Genome Annotation Pipeline and 2755 coding sequences (CDSs) and 84 RNA genes were identified. The G+C content of this genome is 59%. Other databases, including ResFinder server version 2.1 (Available from: https://cge.cbs.dtu.dk/services/ResFinder/), ARG-Annot (Available from: http://en.mediterranee-infection.com/article.php?laref=283%26titre=arg-annot), PlasmidFinder (Available from: https://cge.cbs.dtu.dk/services/PlasmidFinder/), CRISPRFinder (Available from: http://crispr.i2bc.paris-saclay.fr/Server/), Virulence Factors Database (Available from: http://www.mgc.ac.cn/VFs/), PHAge Search Tool (PHAST) (Availble from: http://phast.wishartlab.com/) and BLAST (NCBI) were used for more detailed genome annotation.

**Fig. 1:**
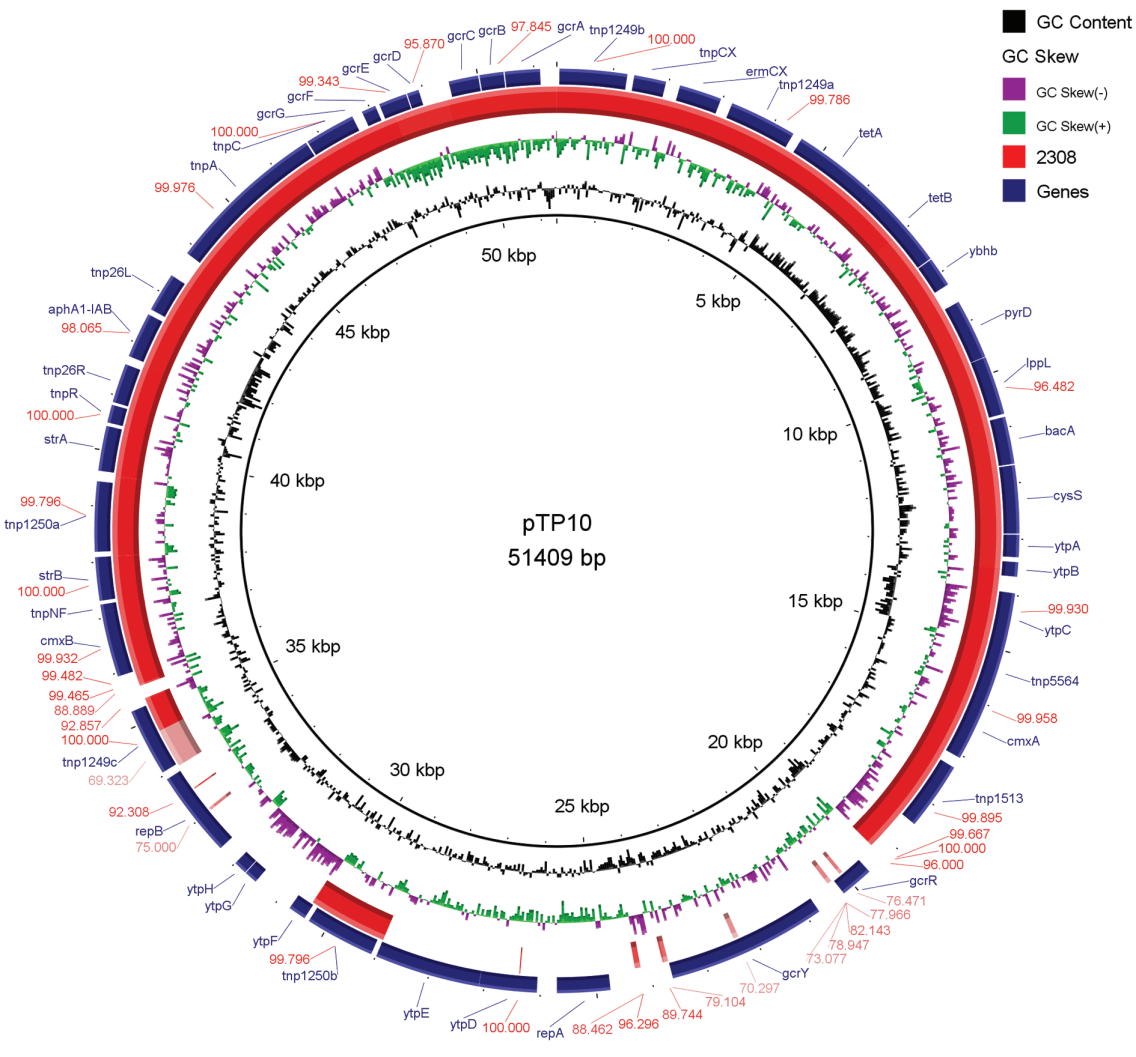
comparison generated by BRIG program using the MDR pTP10 of *Corynebacterium striatum* M82B (Genbank accession number: AF024666) as reference on the inner black circle. Absence of colour in the red ring that represents the genome of MDR *C. striatum* 2308 indicates absence of this region. The *strA-strB* genes are currently called *aph(3”)-Ib-aph(6)-Id.* The gene *aphA1-IAB* gene is currently named *aph(3’)-Ia.*

By phenotypic characterisation, this isolate was susceptible only to tetracycline (MIC 1 mg/L), linezolid (MIC 0,25 mg/L) and vancomycin (MIC 0,5 mg/L). Genotipically, the genome annotation showed the presence of tetA-tetB genes related to the resistance to tetracycline in C. striatum,^12^ ^)^ however our isolate was susceptible to tetracycline. A vanW vancomycin B-type resistance protein copy was found, but until the moment there is no report of resistance to vancomycin in Corynebacterium spp. The resistance to erythromycin (MIC > 256 mg/L) and clindamycin (MIC > 256 mg/L) was associated with the presence of ermX gene inserted near to IS1249 suggesting that a rearrangement of transposon Tn5432 may have occurred.^13^ The presence of the aph(3’)-Ia gene (also known as aphA1) inserted in transposon Tn5715 similar to region of the pTP10 plasmid of C. striatum M82B^13^ (GenBank number: AF024666) may be related to the resistance to the aminoglycoside gentamicin (MIC 256 mg/L), whereas the aph(3”)-Ib-aph(6)-Id genes (also known as strA-strB, respectively) may specifically confer the resistance to aminoglycoside streptomycin (10 µg), confirmed by disk diffusion using values to Staphylococcus spp.^11^

The resistance to quinolones in C. striatum is associated to mutations at codons 87 and 91 of QRDR gyrA gene.^14^ The MIC > 32 mg/L for ciprofloxacin and levofloxacin was related to the mutation at codon 87 of gyrA gene (Ser-87 to Val-87) and no plasmid-mediated quinolone resistance and efflux pumps genes were found. The resistance to penicillin (MIC > 256 mg/L) may be associated to the presence of bla gene with a size of 831pb encoding a class A β-lactamase,^10^ a serine hydrolase belonging to beta lactamase enzyme family with similarity values above 99% with beta lactamases gene sequences from Corynebacterium species deposited in NCBI. Two copies of cmx gene encoding efflux pump to chloramphenicol were found with the IS5564 adjacent to these genes without the IS1513 to form the transposon Tn5564 found in the segment III of plasmid pTP10 C. striatum M82B(13) (GenBank number: AF024666). The resistance to chloramphenicol (30 µg) was confirmed by disk diffusion method using values to Staphylococcus spp.^11^

The PlasmidFinder was used to search replicons of plasmids, however, no replicon was found. So, we use the BLAST Ring Image Generator (BRIG) program^15^ to generate a comparative image between the genome of MDR C. striatum 2308 isolate and the pTP10 plasmid^13^ (GenBank number: AF024666) from multidrug-resistant clinical isolate C. striatum M82B (Fig. 1) which provides genetic information regarding the mechanisms of resistance to 16 antimicrobial agents.^13^ A large part of the genetic content of the pTP10 plasmid^13^ (GenBank number: AF024666) is present in the genome of our isolate, mostly resistance genes, with the exception of the replication machinery associated to mobile genetic elements. To corroborate the absence of replication machinery as part of this element, the reads were mapped against the pTP10 plasmid^13^ (GenBank number: AF024666), recovered and assembled as described elsewhere,^16^ however no replication machinery-related region was found. Amplification of the repB gene by PCR^17^ and the search for the plasmid by PFGE (data not shown) did not show any evidence of the plasmid presence.

**Fig. 2:**
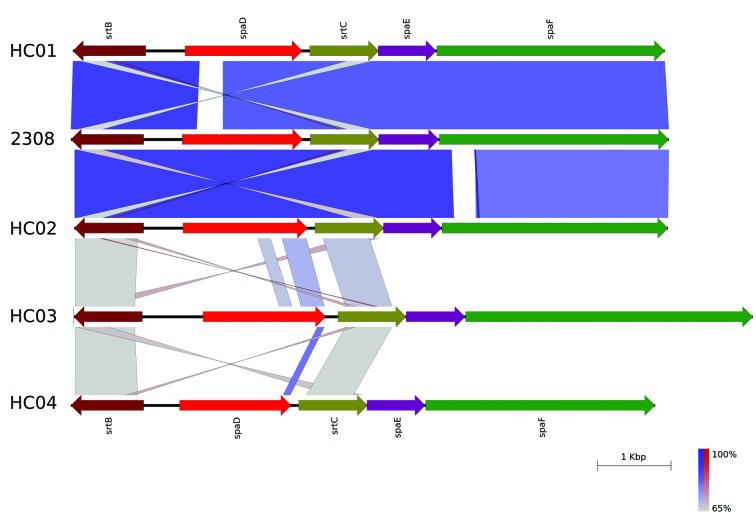
scheme generated by EasyFig program showing the high nucleotide similarity between the *spaDEF* cluster of *Corynebacterium striatum* 2308 isolate and *C. diphtheriae* HC01 (GenBank accession: CP003212) and HC02 (GenBank accession: CP003213). The *C. diphtheriae* HC03 (Genbank accession: CP003214) e HC04 (GenBank accession: CP003215) isolates also were included in the analysis. The *spaDEF* cluster of *C. diphtheriae* HC03 and HC04 is not very similar to *C. diphtheriae* HC01 and HC02 isolates.

Screening for potential virulence factors using the Virulence Factors Database showed the presence of the spaDEF operon that encodes a complete set of pilus proteins and their respective sortases. This cluster was firstly described in Corynebacterium diphtheriae and can play important roles in adhesion to different host tissues. Adhesion to host cells is a crucial step during infection.^18^ ^,^ ^19^ Cell surface pili in Gram-positive bacteria is important to colonisation of host tissues, evasion of the immunity, and the development of biofilms.^20^ The genome organisation of the spaDEF cluster found in C. striatum 2308 isolate is similar to cluster organisation in C. diphtheriae HC01, HC02, HC03 and HC04 (Fig. 2), isolated from cases of endocarditis in Rio de Janeiro, Brazil, with high nucleotide similarity between our C. striatum isolate and C. diphtheriae HC01 and HC02 isolates. However, the SpaA pili proposed as an essential factor in C. diphtheriae to adherence to pharyngeal epithelial cells^21^ was absent in our C. striatum isolate analysed.

Considering that the prophages are important in many bacterial species, including C. diphtheriae where it harbors the tox gene for diphtheria toxin,^18^ we explored the presence of phages in 2308 genome. The prophage regions are unknown in C. striatum and little studied in the genus. A total of four prophage regions have been identified using the PHAST tool in our C. striatum isolate, of which 1 region is incomplete (PHAGE Lactoc1358_NC027120) of 8,7 KB and 3 regions are questionable. Only hypothetical proteins were found and no antibiotic resistance, biofilm formation or virulence genes were visualised.

The CRISPRFinder was used to search clustered regularly interspaced short palindromic repeat (CRISPR), that represents an adaptive and inheritable defense strategy.^22^ In this isolate were found seven CRISPR arrays containing the Cas1, Cas2, Cas3, Cas5 Cas6e, Cas7, Cse1 and Cse2 genes which belong to subtypes I-E in the CRISPR system, one of them associated to IS30 family. The biggest CRISPR array found begins at position 40197 and ends at position 47364 in the contig 18 and has a conserved region GGGCTCATCCCCGCTTACGCGGGGCGGAC (DR length: 29) with 117 spacers. A search against the “My CRISPRs DB” database^23^ enabled us to correlate a part of its sequence to a spacer from bacteria species as Thermofilum pendens, Eubacterium limosum, Roseiflexus castenholzii and others species. These CRISPR regions are important because they confer protection against bacteriophages^19^ and further studies will be carried out.

This report presents the description of some putative mechanisms can be involved in the multidrug-resistance of C. striatum 2308 isolate from a patient with bloodstream infection. The draft genome of this isolate is part of an ongoing study of the genomic analyses and comparison with other clinical isolates to elucidate genetic diversity between them and genetic characterisation of antimicrobial resistance. The whole genome shotgun project has been deposited at Genbank/NCBI under the accession number NRIO00000000.
